# Consequences of geographical accessibility to post-exposure treatment for rabies and snakebite in Africa: a mini review

**DOI:** 10.3389/frhs.2024.1309692

**Published:** 2024-05-30

**Authors:** Aurélia Faust, Nicolas Ray

**Affiliations:** ^1^ GeoHealth Group, Faculty of Medicine, Institute of Global Health, University of Geneva, Geneva, Switzerland; ^2^ Institute for Environmental Sciences, University of Geneva, Geneva, Switzerland

**Keywords:** rabies, snakebite, neglected tropical disease, Africa, accessibility

## Abstract

**Introduction:**

Rabies and snakebite envenoming are two zoonotic neglected tropical diseases (NTDs) transmitted to humans by animal bites, causing each year around 179,000 deaths and are most prevalent in Asia and Africa. Improving geographical accessibility to treatment is crucial in reducing the time from bite to treatment. This mini review aims to identify and synthesize recent studies on the consequences of distance and travel time on the victims of these diseases in African countries, in order to discuss potential joint approaches for health system strengthening targeting both diseases.

**Methods:**

A literature review was conducted separately for each disease using Pubmed, Google Scholar, and snowball searching. Eligible studies, published between 2017 and 2022, had to discuss any aspect linked to geographical accessibility to treatments for either disease in Africa.

**Results:**

Twenty-two articles (8 on snakebite and 14 on rabies) were eligible for data extraction. No study targeted both diseases. Identified consequences of low accessibility to treatment were classified into 6 categories: (1) Delay to treatment; (2) Outcome; (3) Financial impacts; (4) Under-reporting; (5) Compliance to treatment, and (6) Visits to traditional healers.

**Discussion and conclusion:**

Geographical access to treatment significantly influences the burden of rabies and snakebite in Africa. In line with WHO's call for integrating approaches among NTDs, there are opportunities to model disease hotspots, assess population coverage, and optimize geographic access to care for both diseases, possibly jointly. This could enhance the management of these NTDs and contribute to achieving the global snakebite and rabies roadmaps by 2030.

## 1 Introduction

Rabies virus exposures (rabies, hereafter) and snakebite envenoming (snakebite, hereafter) are two zoonotic diseases forming part of the WHOs twenty *Neglected Tropical Diseases* (NTDs) (1). It is estimated that rabies is responsible for 59,000 deaths a year (2), with 25,000 occurring in Africa (3) and 40% of victims being children under 15 years old (1). The disease is transmitted to humans by mammals' saliva, principally by rabid dog bites (1). It is caused by a Lyssavirus (4) that attacks the patient's nervous system leading to severe encephalitis (5). In humans, symptoms develop, on average, after 1–2 months, but may appear after only a few days or, in some cases, several years later (6). Once they have onset, the outcome is almost always fatal (5)*.* However, timely administration of post-exposure prophylaxis treatment (PEP), which includes wound washing, rabies vaccines, and, when necessary, rabies immunoglobulin, can prevent death if administered before the appearance of symptoms (7).

Regarding snakebite, around 2.7 million envenomings are registered each year, resulting in 81,000–138,000 deaths and 400,000 victims being left with a handicap (8). Sub-Saharan Africa accounts for 90,000–300,000 envenomings and 3,500–32,000 deaths (9). Death can occur in few hours for neurotoxic venom, whereas it can take days for other types of venom (hemotoxic, cytotoxic, and myotoxic) (10). Besides mortality, snakebite can lead to amputation, or other handicaps, which may prevent the victim from working and supporting his or her household financially (11). To prevent the effects of venom and enhance the chances of complete recovery, immediate wound cleansing, followed by antivenom administration should be applied after the bite (11).

Mortality and morbidity of these two diseases are likely underestimated due to the high under-reporting of cases (12, 13). The most endemic parts of the world are Asia and Africa (9, 14). Access to healthcare in these areas, particularly in rural zones, is often limited due to their remoteness and challenging geographical features such as mountains, jungles/forests, deserts, or flood-prone areas. Poor infrastructure, and high treatment costs further compound the difficulty in accessing health services (15). The WHO has implemented two roadmaps (2012, 2020) (1, 16) to reduce the impact caused by NTDs. To ameliorate control and prevention of these diseases and aim for the elimination of rabies given the impossibility of eliminating snakebite envenomation, milestones were established. These milestones serve as a guideline for implementing policies and strategies to harmonize practices in concerned countries. For rabies, the goal is to attain zero human deaths from dog-mediated rabies by 2030 (1). For snakebite, the recent dedicated WHO roadmap targets to reduce the number of deaths and cases of disability caused by snakebite by 50% before 2030 (11).

One of the WHO's strategies to boost the efficiency of the fight against NTDs is to “integrate approaches across diseases” (1). Although rabies' need for immediate care is less acute than snakebite, distance to healthcare is a considerable obstacle for timely administration of treatment essential to prevent death and complications for both diseases. Therefore, a conjoint approach might be possible regarding geographical access when putting up strategies to reach the WHO's goals.

A recent study has assessed the practicalities of joint snakebite and rabies control (17). It concluded that one area better suited for inter-disease integrated approaches is health system strengthening, notably through improvement of access to biologicals through joint procurement and strengthened delivery mechanisms. However, the role of geographical accessibility for the various facets linked to post-exposure treatment for both diseases has not been reviewed in a common framework. This mini review aims to fill this gap by identifying and synthesizing the recent existing studies on the consequences of distance and travel time on the victims of each disease in Africa, and by pointing to possible future research in this area.

## 2 Methodology

### 2.1 Eligibility criteria

Publications about geographical accessibility to treatments for snakebites or rabies in African countries were eligible. Articles about non-African countries, animal bite cases, case reports, or studies about diagnosis, management, and treatments of the diseases were excluded. The search was limited to recent publications between January 2017 and March 2022.

### 2.2 Search strategy and screening process

The literature search for rabies and snakebites was done separately by using a series of keywords found in titles or abstracts. Keywords linked to accessibility were the following: “access”, “distance”, “access to treatments”, “travel distance”, and “travel time”. To specify geographical access, we used “geographical”, “geospatial”, and “GIS”. The keywords “health centre”, “healthcare”, “healthcare facilities”, “hospitals”, “dispensaries” were employed to capture healthcare facilities. Finally, the keywords “snakebite”, “snakebite envenoming”, and “rabies” were used for the diseases. Relevant MeSH (Medical Subject Headings) terms were also used. The filter “human” in species was selected to refine the search. The full search queries are available in Supplementary Table S1. PubMed search was complemented by Google Scholar and secondary snowball searches. One author (AF) performs the search and extracted the results. However, eligibility criteria and findings were thoroughly discussed between both authors at all screening and extraction steps. We first screened title and abstracts of articles to exclude non-eligible articles, followed by full text screening of retained articles. Extracted data from retained article included authors, year of publication, country of study, accessibility variable for geographical access, and consequences of geographical access on the impact of diseases.

## 3 Results

**Figure 1:**
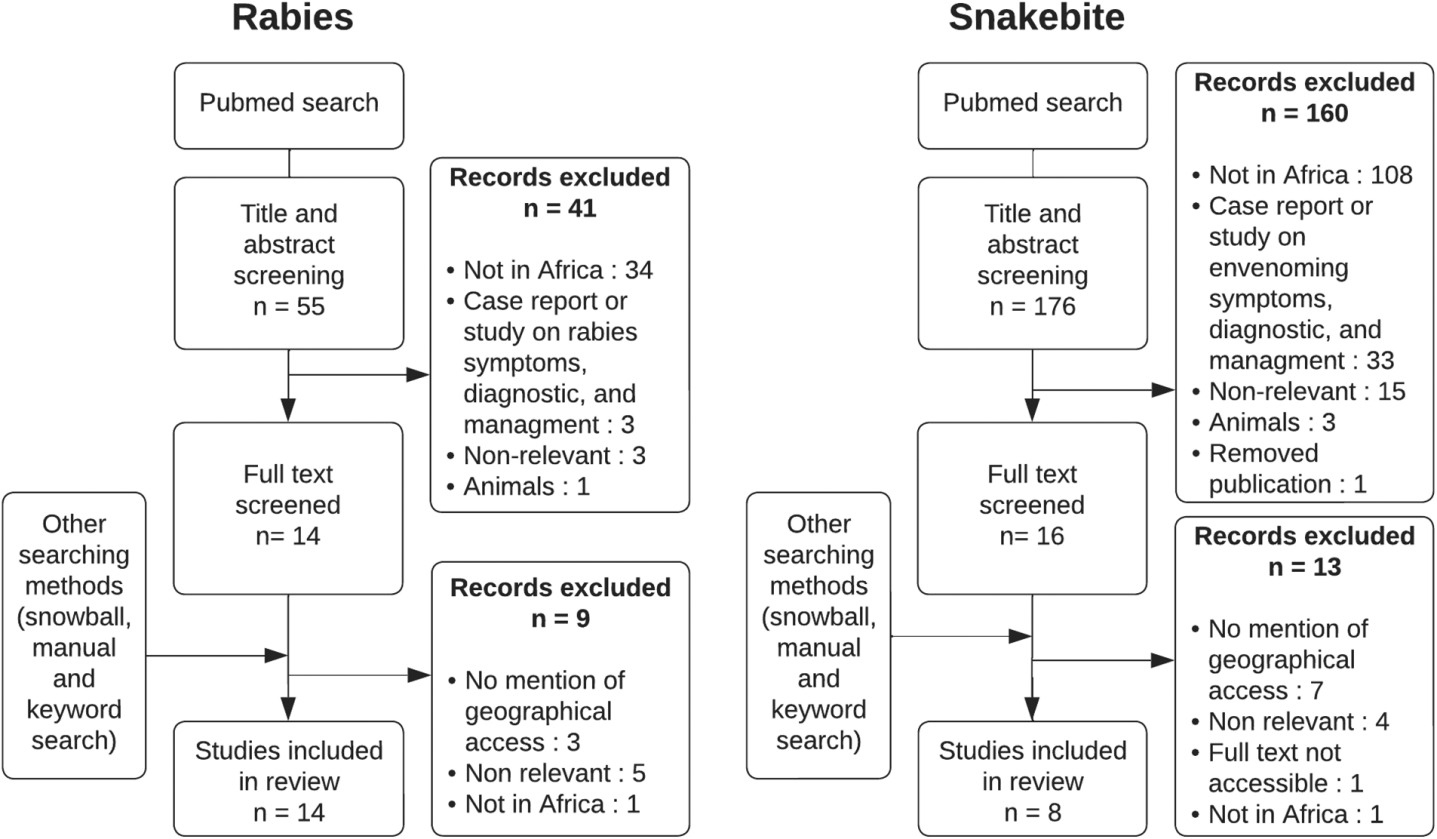
Article selection process for rabies and snakebite.

The literature search resulted in 176 articles on snakebite and 55 on rabies. Twenty-two (snakebites *n* = 8; rabies *n* = 14) articles were selected, out of which fourteen (snakebites *n* = 5; rabies *n* = 9) were identified through the snowball method and Google Scholar. Figure 1 details results for all steps of the article selection process.

Different terms/methods were linked to geographical access. The most used was metrics in km (*n* = 9). Some articles compared rural vs. urban areas to describe accessibility (*n* = 5), while others used travel time (*n* = 2). The remaining six studies did not mention any measuring method, but simply cited “distance” (*n* = 4) or travel cost/transportation (*n* = 2).

The selected studies targeted nine countries. Countries with studies about rabies were: Tanzania (*n* = 4), Ethiopia (*n* = 3), Madagascar (*n* = 2), Cote d'Ivoire (*n* = 2), Uganda (*n* = 1), Ghana (*n* = 1), Kenya (*n* = 1). The studies on snakebite were done in Nigeria (*n* = 3), Kenya (*n* = 2), Tanzania (*n* = 1), Cameroon (*n* = 1) and worldwide (*n* = 1). Supplementary Figure S1 maps these countries.

Among the 22 selected articles, the consequences of geographical access to snakebite and rabies victims were discussed in 18 recent studies whose details are found in Table 1. We classified these studies into six categories that we present below. The remaining four articles addressed poor access but did not explore its impact.

**Table 1:** Records used for data extraction.

Reference	Country	Types of accessibility used or mentioned	Disease	Consequence	Method of selection
van Oirschot et al. (18)	Kenya	Distance	Snakebite	Delay to treatment; Outcome	PubMed
Ochola et al. (19)	Kenya	Mention of distance	Snakebite	Delay to treatment; Traditional healer	PubMed
Longbottom et al. (20)	Global	Travel time	Snakebite	No consequence mentioned	PubMed
Liyasu et al. (21)	Nigeria	Distance	Snakebite	Outcome; Financial; Traditional healer	Snowball
Michael et al. (22)	Nigeria	Mention of distance	Snakebite	Delay to treatment; Outcome; Financial impacts	Snowball
Habib et al. (23)	Nigeria	Mention of distance	Snakebite	Outcome	Snowball
Chuat et al. (24)	Cameroun	Travel cost/transportation	Snakebite	No consequence mentioned	Snowball
Yates et al. (25)	Tanzania	Distance	Snakebite	No consequence mentioned	Snowball
De Nardo et al. (26)	Tanzania	Rural vs. urban	Rabies	Delay to treatment; Compliance	PubMed
Yizengaw et al. (27)	Ethiopia	Rural vs. urban	Rabies	Delay to treatment	PubMed
Rajeev et al. (28)	Madagascar	Travel time	Rabies	Outcome; Underreporting	PubMed
Tetchi et al. (29)	Côte d’Ivoire	Rural vs. urban	Rabies	Underreporting	PubMed
Wangoda et al. (30)	Uganda	Distance	Rabies	No consequence mentioned	PubMed
Changalucha et al. (31)	Tanzania	Distance	Rabies	No consequence mentioned	Snowball
Sambo et al. (32)	Tanzania	Distance	Rabies	Delay to treatment; Outcome; Financial impacts	Snowball
Obonyo et al. (33)	Kenya	Mention of distance	Rabies	Delay to treatment	Snowball
Hampson et al. (34)	Tanzania	Distance	Rabies	Delay to treatment; Outcome	Snowball
Addai et al. (35)	Ghana	Distance	Rabies	Delay to treatment; Compliance	Snowball
Rajeev et al. (36)	Madagascar	Travel costs	Rabies	Underreporting	Snowball
Tschopp et al. (37)	Ethiopia	Urban vs. rural	Rabies	Delay to treatment; Underreporting	Snowball
Beyene et al. (38)	Ethiopia	Distance	Rabies	Compliance	Snowball
Tiembré et al. (39)	Côte d’Ivoire	Urban vs. rural	Rabies	Compliance	Snowball

### 3.1 Delay to treatment

Remote living conditions pose challenges in timely access to healthcare, particularly due to factors such as distance, low quality roads, and transportation costs. For rabies, a Tanzanian study indicated that only 39% (106/272) of patients residing over 10 km from a hospital managed to receive PEP within a week, as opposed to a 64% (92/143) reception rate among closer residents (32). Similar trends were uncovered in retrospective Tanzanian research emphasizing the more likely delay of rural patients in reaching hospitals than their urban counterparts (26). Another study evidenced that 85.7% of those living less than 10 km away reached PEP centers within a week, whereas only 66.2% of more remote dwellers did so (34). Moreover, a study in Ghana revealed a twofold increased likelihood of treatment initiation delay among people being bitten more than 5 km away from a hospital (35). Ethiopian survey data disclosed that the majority of treatments sought after a three-week delay were from rural populations, which were partially due to the fact that there was only one treatment facility for almost 7 million people (27). Another Ethiopian investigation reported treatment commencement up to five days post-bite, predominantly in rural patients (37). A Kenyan retrospective study found that four out of eleven (36%) surveyed patients commenced PEP after more than a two-day delay, with one (1/11, 9%) attributing this to distance to facility (33).

Similar observations were made for snakebite incidences. A study in Niger discovered a delay of over four hours post-bite before reaching treatment in 62.5% (45/72) of patients, 40% (18/72) of whom blamed distance to be the main obstacle (22). A Kenyan study underscored the clinician consensus that patients were unlikely to seek treatment within two hours post-bite due to extended travel distances (19). Another study with 50 participants from Kenya highlighted the challenges of transportation, poor infrastructure, and long distance to hospital in seeking snakebite treatment (18). One patient encapsulated the issue, saying, “Hospitals are far, there are motorbikes, but at the time you don’t have money, so you are forced to walk” (18).

### 3.2 Outcomes

Limited access to healthcare services has been linked to increased mortality rates among rabies victims. In Tanzania, distance from hospitals was found to be a significant predictor of human deaths from rabies. Of the deceased victims following a bite incident, 78% (14/18) resided more than 10 km away from the district hospital, while 89% (16/18) of these fatalities occurred at distance exceeding 60 km away from the regional hospital (32). While not statistically significant, another Tanzanian study found that bite victims developing rabies tended to reside farther away from hospitals compared to those who did not develop the disease (34). An increase in death incidence with travel time to the clinic was also observed in Madagascar (28).

For snakebite, the association between distance and severe outcomes or deaths was less clear. A study in Kenya reported the death of a girl before reaching a hospital located 25 km away (18). A retrospective study in Nigeria found a significant relationship between delay and mortality, with 55% (52/94) of deaths occurring in patients outside Gombe, where the hospital was situated. The study also showed that for every hour of delay, there was 1% increase in the odds of mortality (23). In a study in Nigeria, although distance and delay were not significantly correlated to bad outcomes, out of the 6 patients who died, 4 (67%) of them lived more than 100 km away. The study also reported an increase of 2% of the risk of bad outcomes for every hour delay (21). Another Nigerian study found no association between mortality and late arrivals at the clinic, but noted a higher (3x) odds of wound infection in patients with delayed presentations (22).

### 3.3 Financial impacts

Distance to healthcare can have financial consequences for bite victims. In Tanzania, bite victims residing in rural areas farther from the hospital incurred higher direct and indirect expenses compared to urban residents due to the costs associated with traveling longer distances for rabies treatment (32). For snakebite victims, increased distance and delay in seeking medical attention resulted in the venom spreading further in the body and leading to more severe infections. In Nigeria, snakebite patients arriving at the hospital after 4 h had to pay a median hospitalization cost about twice as large as the median cost for those arriving earlier. Additionally, patients arriving late often required a larger dose of antivenom (22). Another study in Nigeria also observed that patients bitten at greater distances required multiple shots of antivenom (179.4 km vs. 136.9 km) (21).

### 3.4 Under-reporting of cases

Accessibility to healthcare facilities directly impacts the reporting rate of bite incidents. An under-reporting of cases can occur when victims find it difficult or impossible to reach a treatment center. In Madagascar, a study found that the incidence of dog bites increased with shorter travel times (28). In the same country, 8 out of 17 patients (47%) cited lack of travel funds as the reason for not seeking medical help after a dog bite (36). In Cote d'Ivoire, 87% (1,099/1,263) of patients attending anti-rabies facilities were from urban areas due to the significant distances of hospitals in rural areas (29). A study in Ethiopia found that the primary reasons for patients not seeking care in healthcare facilities after dog bites were the distance and logistical challenges involved in getting to these facilities (37). Consequently, this lack of healthcare seeking behavior contributes significantly to the under-reporting of these diseases.

### 3.5 Visit to traditional healer after a snakebite

Populations residing long distances from healthcare facilities often visit traditional healers when bitten by a snake. A Kenyan professional deemed the lack of accessibility as the main reason for patients opting for traditional healers first (19). Similarly, a Nigerian study noted a pattern where victims bitten at locations over 100 km from medical centers were more likely to consult a traditional healer first [252/273 (92.3%) vs. 102/124 (82.3%)] (21). People interviewed in Cameroon reported that they would turn to traditional healers if the travel distance and costs involved in reaching a hospital were too high (24). One woman further explained that, if all conditions were favorable (financial resources and transportation), she would seek help from a hospital immediately after a snakebite. Otherwise, she would choose a traditional healer (24).

### 3.6 Compliance to treatment for rabies

Completing rabies prophylaxis involves multiple vaccine doses, which can be difficult for patients who face challenges in accessing healthcare facilities. An Ethiopian study highlighted a negative correlation between the distance from a hospital and the likelihood of seeking treatment. Each kilometer closer increased the probability of treatment completion by 4% (38). A Tanzanian research study found that rural populations are statistically more likely to fail to complete treatment and incur loss of follow-up (26). In Cote d'Ivoire, populations outside of Abidjan had significantly lower treatment adherence, with 81.7% (94/115) failing to complete treatment compared to 37% (156/418) in the city (39). Interestingly, a study in Ghana did not find a significant association between travel distance and PEP completion (35).

## 4 Discussion

The 18 articles included in our review of recent studies have demonstrated the significant impact of geographical accessibility to treatment in relation to snakebite and rabies in Africa, although no studies addressed both diseases jointly. However, certain aspects of accessibility were found to be common to both diseases such as delay to treatment, outcomes, and financial consequences. While distance to healthcare facilities might create challenges in accessing timely treatment, it is not the only determinant of poor outcomes for individuals living farther away from healthcare services. Indeed, better-resourced areas near healthcare facilities may benefit from enhanced bite prevention education and community awareness efforts, which could be lacking in remote regions.

The role of physical accessibility for compliance to treatment was only discussed in studies on rabies. This finding is expected since PEP requires multiple visits to the healthcare facilities, unlike antivenom treatment for snakebite. Visits to traditional healers was only found to be linked to snakebite, which increased with distance from facilities providing snakebite treatment. However, this observation could be due to our search criteria and the restricted range of our search. Additionally, a recent global review revealed that traditional healers are also visited for rabies prevention, notably in Nigeria and Ethiopia (40).

Furthermore, our review found several studies that concluded under-reporting of dog bite cases is associated with the distance to treating facilities. Accurately estimating the burden of snakebite and rabies, along with its geographical distribution, is essential to improve availability and access to treatment. Unfortunately, such estimations are often lacking, which partly explains the lack of engagement from some governments and health organizations in addressing these two NTDs (23, 41, 42). Running national household surveys, rather than relying solely on hospital-based surveys, is a solution to under-reporting, as recently done for snakebite in Nepal (43). However, this approach is costly and time-consuming. Correcting under-reporting by considering the distance to care has been recently proposed for addressing the dog bites burden in Madagascar (28) and the Dengue incidence in the Philippines (44). To our knowledge, no such correction framework exists for snakebite burden estimation, which could be an area of future research.

However, the link between distance and bite reporting does not always imply causation, as even communities with healthcare access may underreport cases. Education and awareness, among the public and healthcare workers, significantly influence the reporting of bites and its impact to authorities. Limited awareness of rabies and snakebite dangers can prevent seeking help. Moreover, healthcare staff recognizing and reporting cases is crucial. A community-based awareness program showed great success in Nepal in increasing snakebite reporting and could be done in Africa too (45).

Another important factor contributing to treatment delay is the unavailability of appropriate treatments. Patients often have to visit multiple healthcare facilities in the hope of receiving treatment and may be required to travel long distances (18, 33). Even when treatment is available, delays may occur due to untrained staff (46). Consequently, it is crucial to have a comprehensive inventory of all healthcare facilities that stock treatments and to keep this information updated for effective health system planning. The WHO is currently implementing the “Snakebite Information and Data Platform” initiative, which aims to achieve this goal for snakebite (47). Additionally, establishing a system where health professionals can report shortages of treatments at their clinics using mobile phones, as described in a study on rabies in Tanzania (48), would help maintain an accurate registry. Bite victims could call a hotline and be directed to the nearest facility with available treatments, as previously implemented in Chad (49). To optimize this system for snakebite, it could be combined with volunteer-based motorcycle transportation, which has shown success in Nepal by facilitating the rapid transport of victims to the nearest facility with treatment (45). This would not only reduce time to treatment but would also reduce overall costs by decreasing travel costs and diminishing financial impact of complications and disabilities. Additionally, training staff to adhere to WHO and national guidelines could prevent treatment overuse, ultimately reducing patients' expenses.

In many regions of Africa, lack of awareness regarding the dangers and consequences of snakebite (18) and ignorance surrounding the existence and transmission of rabies (36) have been observed. However, information campaigns have significantly improved in recent years, resulting in increased awareness of the importance of rapid access to treatment. Traditional practitioners are often consulted after a bite. Engaging them in addressing the impacts of rabies and snakebites by teaching first aid methods and emphasizing the importance of hospital referrals (24) could enhance outcomes and reduce delays. Such collaboration has already been shown to be efficient in Nepal for snakebites (45).

Many studies in our review highlighted the similarities in the shortcomings of accessibility to treatment for both diseases. Considering this critical importance of timely access to treatment, the use of geospatial approaches to model disease hotspots, assess population coverage and optimized geographic access to care holds great promise for joint approaches on rabies and snakebite in Africa. Recent modeling of global snakebite hotspot has been conducted (20), and higher resolution models have been developed in Nepal (50). Least-cost path models are particularly well-suited for low-resource settings where patients use a combination of walking and other modes of transportation to reach healthcare (51, 52). These models have been used to quantify population access to antivenom and propose optimized scenario for improvement in Costa Rica (53) and Nepal (54). In an earlier review (55), we showed that infectious diseases with an acute need for treatment, such as rabies, were particularly underrepresented in the literature covering spatial accessibility. Therefore, integrating accessibility modeling data with rabies and snakebite hotspot maps could help identify regions with high burdens but inadequate access to antivenom and PEP. Consequently, governments could allocate resources accordingly to address these regions effectively. Additionally, delivering both treatments together in areas affected by both diseases could optimize cold chain installations and reduce costs (17). As the quality and accessibility of geospatial data continue to improve in many African countries, it is crucial to encourage the implementation of these modeling approaches along with national epidemiological studies on both rabies and snakebite burdens.

Our mini review was limited by the use of PubMed for the previous five years. Despite conducting manual snowball searches to identify key literature, we may have missed recent publications. Our mini-review emphasized the substantial role of geographic accessibility, or the lack thereof, in various aspects related to the treatment and outcomes of snakebite and rabies. There is a strong potential for future research and implementation studies to consider a joint approach in addressing access to PEP and antivenom. In line with the WHO's call for integrating approaches among NTDs, such a joint approach could contribute to achieving the global snakebite and rabies roadmaps by 2030.
