# Supplementary material for: Consequences of geographical accessibility to post-exposure treatment for rabies and snakebite in Africa: a mini review
Source: Front Health Serv. 2024 May 30;4:1309692. doi: 10.3389/frhs.2024.1309692 (PMC11169726; doi:10.3389/frhs.2024.1309692)
Supplement: Supplementary file 1 [file Datasheet1.docx]

**Supplementary Figures and Tables**

Tables

Supplementary Table 1. Search queries used in PubMed

| **Snakebites** | (access*[Title/Abstract] OR distance*[Title/Abstract] OR "access to treatments"[Title/Abstract] OR "travel distance"[Title/Abstract] OR "travel time"[Title/Abstract] OR "Health Services Accessibility"[Mesh]) AND (geograph*[Title/Abstract] OR geospatial[Title/Abstract] OR GIS[Title/Abstract] OR "Geographic Mapping"[Mesh] OR "Geographic Information Systems"[Mesh]) AND ((health[Title/Abstract] AND (center*[Title/Abstract] OR care*[Title/Abstract] OR facilit*[Title/Abstract]))) OR (hospital*[Title/Abstract] OR dispensar*[Title/Abstract]) AND (snakebite*[Title/Abstract] OR "snake bite*"[Title/Abstract] OR "snake envenom*"[Title/Abstract]) |
| --- | --- |
| **Rabies** | (access*[Title/Abstract] OR distance*[Title/Abstract] OR "access to treatments"[Title/Abstract] OR "travel distance"[Title/Abstract] OR "travel time"[Title/Abstract] OR "Health Services Accessibility"[Mesh]) AND (geograph*[Title/Abstract] OR geospatial[Title/Abstract] OR GIS[Title/Abstract] OR "Geographic Mapping"[Mesh] OR "Geographic Information Systems"[Mesh]) AND ((health[Title/Abstract] AND (center*[Title/Abstract] OR care*[Title/Abstract] OR facilit*[Title/Abstract]))) OR (hospital*[Title/Abstract] OR dispensar*[Title/Abstract]) AND ("human rabies"[Title/Abstract] OR "Rabies"[Mesh]) |
| **Filters** | Species: Human |

**Figures**

Supplementary Figure 1. Countries where selected studies were conducted for rabies (A) and snakebite (B).

**(A)**


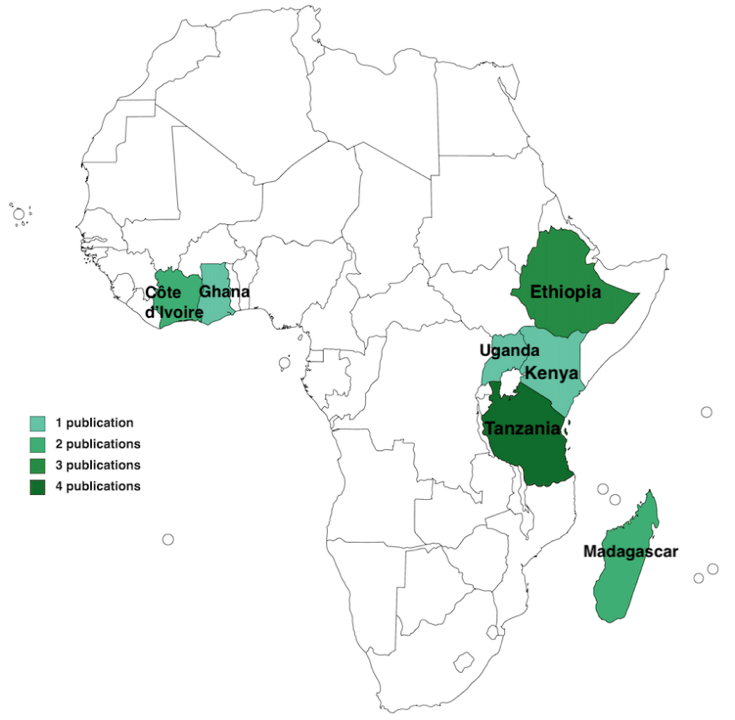


**(B)**


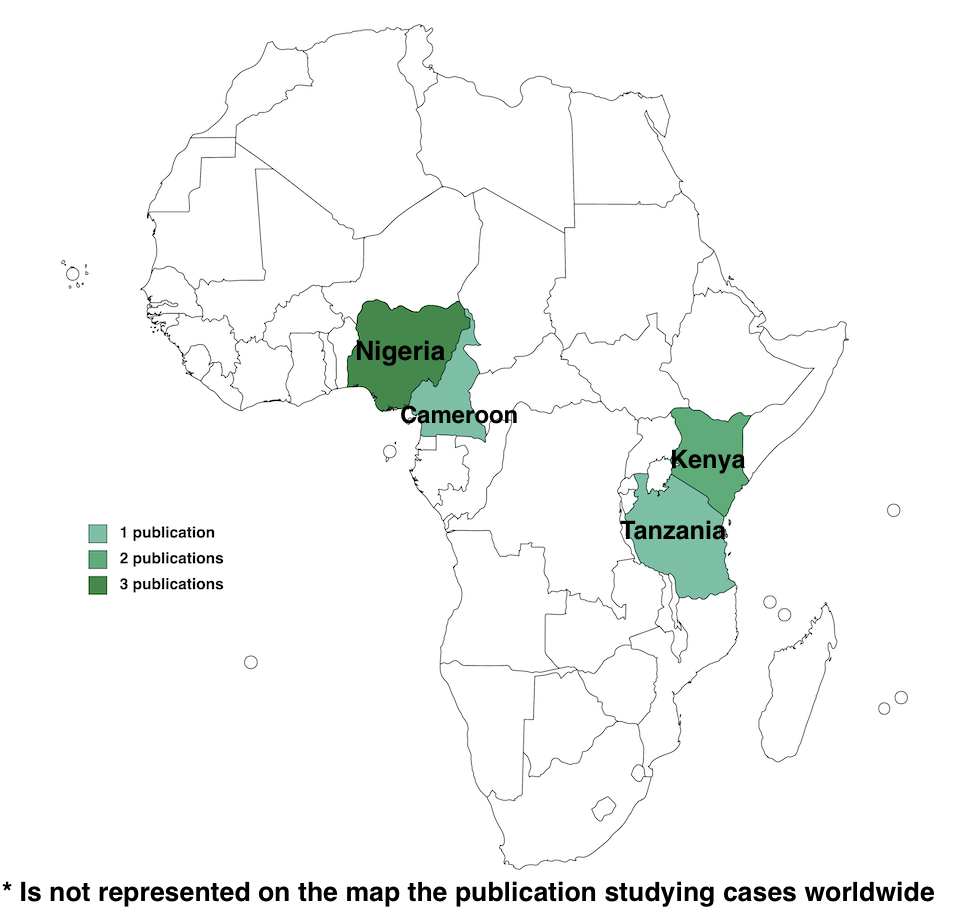

One selected global study on vulnerability to snakebite is not represented on the map.
